# Sexual Orientation and Cognitive Ability: A Multivariate Meta-Analytic Follow-Up

**DOI:** 10.1007/s10508-020-01632-y

**Published:** 2020-01-23

**Authors:** Yin Xu, Sam Norton, Qazi Rahman

**Affiliations:** Department of Psychology, Institute of Psychiatry Psychology and Neuroscience, King’s College London, 5th Floor Bermondsey Wing, Guys Hospital Campus, London, SE1 9RT UK

**Keywords:** Sexual orientation, Meta-analysis, Sex differences, Cognition, Spatial, Verbal

## Abstract

A cross-sex shift model of human sexual orientation differences predicts that homosexual men should perform or score in the direction of heterosexual women, and homosexual women in the direction of heterosexual men, in behavioral domains such as cognition and personality. In order to test whether homosexual men and women’s cognitive performance was closer to that of heterosexual men or that of heterosexual women (i.e., sex-atypical for their sex and closer to that of the opposite-sex), we conducted a multivariate meta-analysis based on data from our previous meta-analysis (Xu, Norton, & Rahman, 2017). A subset of this data was used and comprised 30 articles (and 2 unpublished datasets) and 244,434 participants. The multivariate meta-analysis revealed that homosexual men were sex-atypical in mental rotation (Hedges’ *g* = −0.36) and the water level test (Hedges’ *g* = −0.55). In mental rotation, homosexual men were in-between heterosexual men and women. There was no significant group difference on spatial location memory. Homosexual men were also sex-atypical on male-favoring spatial-related tasks (Hedges’ *g* = −0.54), and female-favoring spatial-related tasks (Hedges’ *g* = 0.38). Homosexual women tended to be sex-typical (similar to heterosexual women). There were no significant group differences on male-favoring “other” tasks or female-favoring verbal-related tasks. Heterosexual men and women differed significantly on female-favoring “other” tasks. These results support the cross-sex shift hypothesis which predicts that homosexual men perform in the direction of heterosexual women in sex differentiated cognitive domains. However, the type of task and cognitive domain tested is critical.

## Introduction

Recently, we conducted a meta-analysis to test the relationship between sexual orientation and cognitive performance on tasks that show normative sex differences (Xu, Norton, & Rahman, 2017). This was motivated by the cross-sex shift model of sexual orientation differences which predicts that homosexual men should behave more like heterosexual women than heterosexual men do, and homosexual women behave more like heterosexual men than heterosexual women do, in sex differentiated domains such as cognitive ability. The pattern of effect sizes found (ranging from small to medium) appeared to support the notion that homosexual men are cross-sex shifted on both male-favoring (e.g., mental rotation) and female-favoring tasks (e.g., verbal fluency). Homosexual women appeared cross-sex shifted only on male-favoring tasks (a small effect size). Cognitive domain affected the magnitude of the differences. For example, studies testing group differences in spatial-related task domains revealed the largest effect size in men.

These findings may be important for causal models of sexual orientation development, such as the prenatal androgen theory. This theory predicts that homosexuals of both sexes should perform, score or otherwise behave in the same direction as their opposite-sex heterosexual peers in behavioral domains where sex differences are typically found. This is hypothesized to be due to the actions of prenatal sex hormones upon developing brain mechanisms underlying both sexual orientation and its behavioral correlates (Ellis & Ames, 1987; Rahman, 2005a). Prenatal sex hormones may organize both sexual orientation and cognitive ability in sex-atypical directions in homosexual men and women. Several lines of evidence support this hypothesis (reviewed in Bailey et al., 2016; see also Hines, Brook, & Conway, 2004; Mueller et al., 2008; Puts, McDaniel, Jordan, & Breedlove, 2008). This does not exclude the possible role of other factors, such as learning and gender-related experiences, although evidence for these in relation to sexual orientation cognitive differences is lacking. Importantly, while prenatal androgen theory suggests that prenatal periods may be important, there may, in fact, be more than one critical period for males, and more sensitive periods for females, during which sex hormones act (McCarthy, Herold, & Stockman, 2018). It is also possible that sexual variation in behavioral and cognitive outcomes is influenced by environmental factors after the critical period and possibly around puberty (Koss & Frick, 2017).

Since our original meta-analysis was published, we have received feedback from scholars in the fields of sex research, psychology, and cognition regarding how “shifted” homosexual men and women’s cognitive performance is directly compared with heterosexual comparison groups. In other words, were homosexual men and women’s cognitive performances closer to that of heterosexual men or that of heterosexual women? In our original meta-analysis, we averaged the difference between homosexual and heterosexual men, or between homosexual and heterosexual women across various cognitive performance types. We did not directly compare homosexual men with heterosexual women, or compare homosexual women with heterosexual men. Thus, our effect sizes could not tell us whether homosexual men or women’s cognitive performances were closer to that of heterosexual men or that of heterosexual women. However, they could be clearly inferred from the patterns reported and by comparing those to prior meta-analytic findings concerning normative sex differences in the relevant cognitive task or domain (e.g., Hyde & Linn, 1988; Voyer, Voyer, & Bryden, 1995). Naturally, this approach is limited because prior research on sex differences and our meta-analysis examined different samples. As part of the on-going discussion and post-publication peer review regarding our study, here we present the results of a new multivariate meta-analysis to help answer the question of the directionality in sexual orientation-related cognitive differences.

A multivariate meta-analysis would allow us to directly test whether homosexual men and women’s cognitive performances were closer to that of heterosexual men or that of heterosexual women. Multivariate meta-analysis is a generalization of univariate meta-analysis, which has a wide range of applications and is often used to analyze data where effect sizes represent group differences across different constructs (e.g., multiple correlated outcomes) or multiple different groups on single outcomes (see Jackson, Riley, & White, 2011). An example of the latter is where we wish to estimate the comparative effectiveness of different treatments even where head-to-head trials are not available due to having common control groups. By way of illustration, consider two treatments A and B used to treat a disease that has been compared only to a control treatment C in clinical trials. In order to estimate the relative efficacy of treatment A versus treatment B, we have only indirect evidence based on the difference in efficacy of treatments A and B compared to treatment C. Instead of conducting two pairwise univariate meta-analyses (comparing A vs. C and B vs. C, separately), a multivariate “network” meta-analysis allows us to pool information from the direct comparisons observed in the literature to also estimate effect sizes for the indirect comparison not observed, along with standard errors, confidence intervals, and *p* values (Salanti, 2012).

In our case, we can use this approach to examine the relative difference in cognitive performance among four groups (heterosexual men as the reference group, heterosexual women, homosexual men, and homosexual women). Essentially, we have the same situation as in a network meta-analysis except that instead of pooling treatment group differences against a common control condition we can pool group differences against the mean for heterosexual males. This allows us to put the effect sizes for the group differences on a common metric, which enables comparisons of the relative performance across all groups. That is, we are able to estimate on a continuum not just how “shifted” homosexual men and women’s cognitive performance is directly compared with heterosexual comparison groups but also relative to each other.

The objective of current research was to directly test whether homosexual men and women’s cognitive performances were closer to that of heterosexual men or that of heterosexual women via a multivariate meta-analysis. To do this, we use a subset of the data available in our previous meta-analysis. We include tests for the effects of specific spatial tasks which have been most intensively studied (mental rotation, the water level test, and spatial location memory). We also examine cognitive domain (male-favoring, female-favoring, spatial, verbal, and other). Note we did not test for other moderators (age, education level, and exclusivity of sexual orientation) because these showed no or very small effects in our prior meta-analysis.

## Method

The details of our methods used to select eligible articles, code moderating variables and compute effect size are described in our prior meta-analysis (Xu et al., 2017). When studies used multiple tests for the same cognitive performance type, we selected the most commonly used test across studies to compute effect size since these studies did not provide the correlations among outcomes. This resulted in the reduced data size used here because we can only analyze one outcome per study using this statistical approach (and makes our approach somewhat different to a typical network-type, multiple treatment meta-analysis).

The multivariate meta-analysis was performed using Stata 15.0. We followed the instructions suggested by prior research (White, 2015). Separate multivariate random-effects meta-analyses were conducted using the package *mvmeta* for eight cognitive test groupings (White, 2011). Models were estimated using restricted maximum likelihood under the assumption of consistency with sex and sexual orientation groups included as dummy variables where heterosexual males were the reference group. Effects sizes were expressed as standardized mean differences calculated as Hedges’ *g* with a correction for the known upward bias in small samples.

The cognitive test groupings included the three most commonly measured cognitive tests with traditionally larger effect sizes and most studied in the field (mental rotation test, water level test, and spatial location memory) and five cognitive domain types (male-favoring spatial-related tasks, male-favoring other tasks, female-favoring spatial-related tasks, female-favoring verbal-related tasks, and female-favoring other tasks). Male-favoring spatial-related tasks are defined as spatial tasks on which heterosexual men outperform heterosexual women on average, including mental rotation, spatial perception, spatial visualization, spatial orientation, and spatial learning/navigation. Male-favoring other tasks include the dichotic listening test. Female-favoring spatial-related tasks are defined as those on which heterosexual women outperform heterosexual men on average, including object location memory. Female-favoring verbal-related tasks are defined as those on which heterosexual women outperform heterosexual men on average, including verbal and semantic fluency. Female-favoring other tasks included perceptual speed and facial emotion recognition.

## Results

**Fig. 1:**
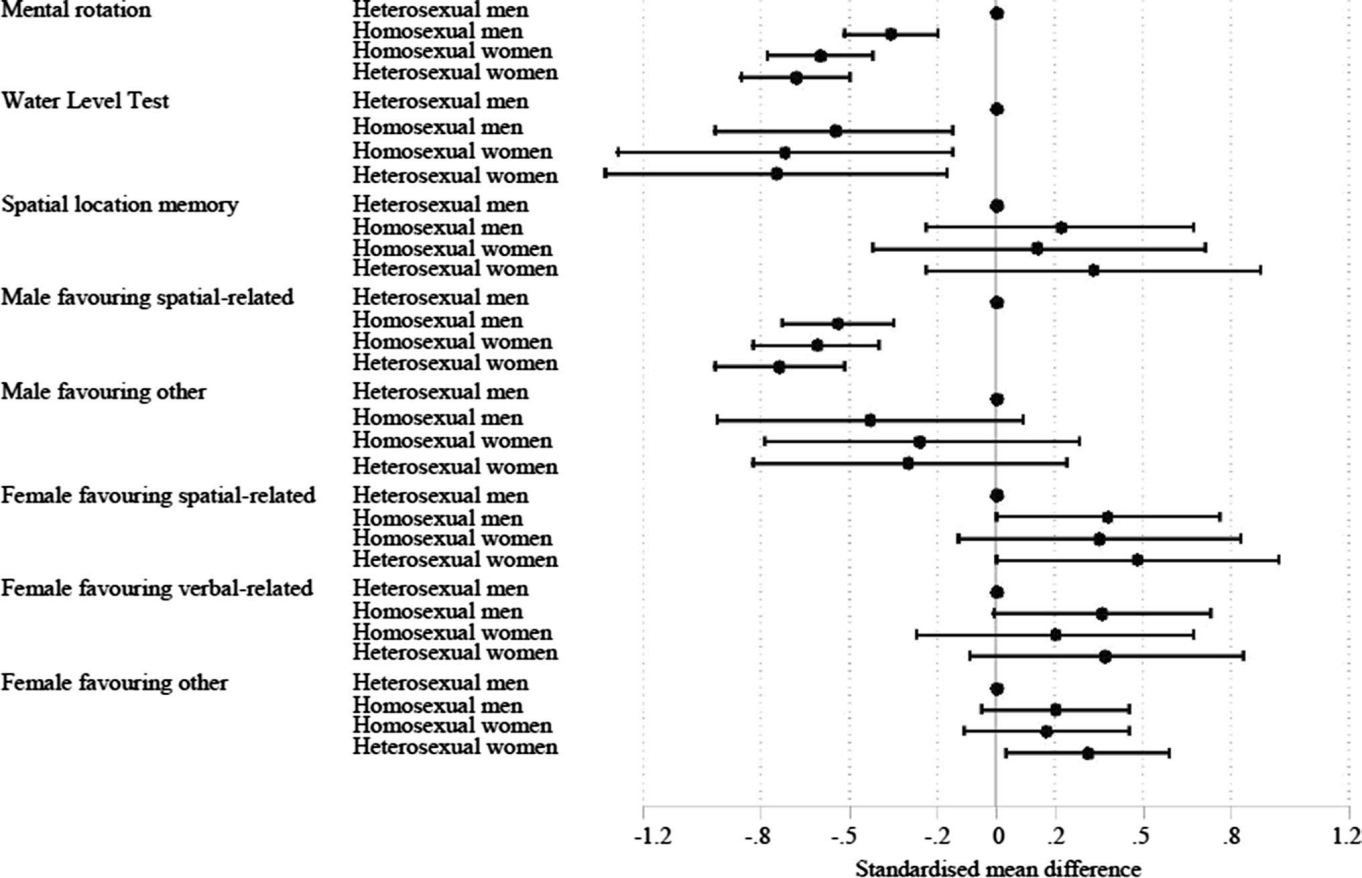
The pooled effect size (Hedges’ *g*) separately by specific cognitive tests and cognitive performance types

Table 1 shows the numbers of studies and participant numbers included in the multivariate meta-analysis, separately by specific cognitive test and cognitive domain types. Table 2 and Fig. 1 show the pooled effect size by specific cognitive tests. Homosexual men’s performance on these three tests was shifted in the direction of heterosexual women and was closer to that of heterosexual women than that of heterosexual men. Heterosexual men significantly outperformed homosexual men on mental rotation and water level tests, Hedges’ *g* = −0.36, *Z* = −4.51, *p* < .001 and Hedges’ *g* = −0.55, *Z *= −2.67, *p* < .01. There were no statistically significant differences in spatial location memory. On mental rotation, homosexual men were equidistant between heterosexual men and women. Homosexual women’s performance on these cognitive tests was closer to that of heterosexual women than that of heterosexual men.

**Table 1:** Numbers of studies and participant numbers in the multivariate meta-analysis, separately by specific cognitive tests and cognitive performance types

Variable	*K*	*N*
Mental rotation test	13	129,928^a^
Water level test	6	567
Spatial location memory test	4	195,928^a^
Male-favoring, spatial-related	20	242,956^a^
Male-favoring, other	4	318
Female-favoring, spatial-related	6	196,048^a^
Female-favoring, verbal-related	9	196,471^a^
Female-favoring, other	5	707

*K* number of studies

^a^The large number of participants is due to the inclusion of the BBC SexID study

**Table 2:** The pooled effect size (Hedges’ *g*) separately by specific cognitive tests on which the largest number of studies have been conducted

Group	Effect size (95% CI)					
	*N*^a^	Mental rotation	*N*	Water level test	*N*^a^	Spatial location memory
Heterosexual men	121,565	0 (reference group)	189	0 (reference group)	97,843	0 (reference group)
Heterosexual women	109,377	− 0.68*** (− 0.87, − 0.50)	93	− 0.75* (− 1.33, − 0.17)	79,176	0.33 (− 0.24, 0.90)
Homosexual men	7799	− 0.36*** (− 0.52, − 0.20)	197	− 0.55** (− 0.96, − 0.15)	10,570	0.22 (− 0.24, 0.67)
Homosexual women	3757	− 0.60*** (− 0.78, − 0.42)	88	− 0.72* (− 1.29, − 0.15)	8339	0.14 (− 0.42, 0.71)

*95% CI* 95% confidence interval

^a^The large participant numbers here are driven by the BBC SexID study sample

*p < .05; **p < .01; ***p < .001

Table 2 and Fig. 1 also show the pooled effect size by cognitive domain. Again, there was a clear tendency for homosexual men’s performance to be closer to that of heterosexual women than that of heterosexual men. Heterosexual men outperformed homosexual men on male-favoring spatial-related tasks, Hedges’ *g* = −0.54, *Z *= −5.52, *p* < .001, while heterosexual men performed lower than homosexual men on female-favoring spatial-related tasks, Hedges’ *g* = 0.38, *Z* = 1.97, *p* < .05. There were no significant group differences on male-favoring other tasks or female-favoring verbal-related tasks. Heterosexual men and heterosexual women differed significantly on female-favoring other tasks. Again, homosexual women were similar to heterosexual women on each cognitive domain. In general, we can see a clear ordering for tasks that are male favoring with homosexual men performing closer to heterosexual women than heterosexual men, but still not as close as the homosexual women. The ordering for the female-favoring tasks was less patterned (Table 3).

**Table 3:** The pooled effect size (Hedges’ *g*) separately by cognitive domain types

Group	Effect size (95% CI)									
	*N*^a^	Male-favoring, spatial related	*N*	Male-favoring, other	*N*^a^	Female-favoring, spatial-related	*N*^a^	Female-favoring, verbal-related	*N*	Female-favoring, other
Heterosexual men	121,705	0 (reference group)	80	0 (reference group)	97,883	0 (reference group)	98,045	0 (reference group)	199	0 (reference group)
Heterosexual women	109,464	− 0.74*** (− 0.96, − 0.52)	80	− 0.30 (− 0.83, 0.24)	79,196	0.48^b^ (0.00, 0.96)	79,245	0.37 (− 0.09 0.84)	154	0.31* (0.03 0.59)
Homosexual men	9821	− 0.54*** (− 0.73, − 0.35)	82	− 0.43 (− 0.95, 0.09)	10,610	0.38* (0.00, 0.76)	10,773	0.36^c^ (− 0.01, 0.73)	200	0.20 (− 0.05, 0.45)
Homosexual women	11,389	− 0.61*** (− 0.83, − 0.40)	76	− 0.26 (− 0.79, 0.28)	8359	0.35 (− 0.13, 0.83)	8408	0.20 (− 0.27, 0.67)	154	0.17 (− 0.11, 0.45)

*95% CI* 95% confidence interval

*p < .05; **p < .01; ***p < .001

^a^The large participant numbers here are driven by the BBC SexID study sample

^b^*p* = .052

^c^*p *= .057

## Discussion

This analysis produced three main findings. First, homosexual men were sex-atypical in studies measuring mental rotations, the water level test, male-favoring spatial-related tasks, and female-favoring spatial-related tasks. That is, homosexual men’s cognitive performance was closer to that of heterosexual women than heterosexual men. Second, homosexual women were no different to heterosexual women, despite some tendency to be sex-atypical in certain domains (e.g., female-favoring verbal-related tasks). Third, there was considerable heterogeneity in the data as we found in our original meta-analysis.

The magnitude of the effect sizes revealed in the current multivariate meta-analysis was similar to that of our prior univariate meta-analysis. Once again, we found that homosexual men showed a cross-sex shift in male- and female-favoring spatial tasks, which is consistent with our prior demonstration that effect size was the highest for spatial tasks in men (Xu et al., 2017). The results for women were also consistent with previous work, suggesting that homosexual women are by and large sex-typical in most cognitive domains. However, given that the studies included in the current multivariate meta-analysis are a subsample of those from our prior study, the reduced number of studies may have contributed to the non-significant results found in women.

Our results should not be interpreted as indicating that homosexual men performed exactly the same as heterosexual women. In other words, we find little evidence of a complete sex inversion in this behavioral domain among homosexual men. Task type and cognitive domain are clearly critical. Traditionally, male-favoring spatial tasks (particularly mental rotation and spatial relations) appear to be most sensitive to sexual orientation differences. This is most likely due to the fact that they show robust general sex differences (Voyer et al., 1995) and that this domain provided the greatest number of studies. The cross-sex shifted pattern displayed by homosexual men is consistent with that found in several other behavioral domains such as sex-typed behavior and personality (Bailey et al., 2016). However, the effect sizes found here are much smaller than for other traits associated with sexual orientation, such as childhood gender nonconformity (Bailey et al., 2016).

In general, the body of work supports the prenatal androgen theory which predicts that homosexual men should show cross-sex shifts in sex differentiated behavioral domains in line with the atypical shift in their sexual partner orientation (Ellis & Ames, 1987). As the present study did not directly measure prenatal androgen levels, caution must be exercised in interpretation. However, some remarks regarding the patterns reported here and their relationship to the prenatal androgen model are worthwhile. The evidence for a cross-sex shift in cognition is inconsistent with research using putative markers of prenatal androgen exposure. For example, digit ratio (2D:4D) is a marker ascribed to the actions of prenatal androgen levels. However, nonheterosexual women have more masculine digit ratios (indicating greater exposure to prenatal androgens) than heterosexual women, but there is no significant difference in digit ratios between heterosexual and nonheterosexual men (Grimbos, Dawood, Burriss, Zucker, & Puts, 2010). Similarly, differences in handedness are a feature sometimes ascribed to the actions of prenatal testosterone acting on developing brain asymmetries. However, both nonheterosexual men and women are significantly more likely to be non-right-handed than heterosexual men and women rather than cross-sex shifted (Lalumière, Blanchard, & Zucker, 2000). As mentioned earlier, sexual orientation-related differences in sex-typed behavior (e.g., play and peer preferences), personality, and sexual orientation target preference itself (the preference for males or females as sexual and romantic partners) are much larger than cognitive differences (Bailey et al., 2016). Some of these traits (sex-typed behaviors) may show substantially larger sex and sexual orientation-related differences during childhood than other traits (cognition). Thus, it is possible that these discrepant findings where some traits show cross-sex shifts (cognition, sex-typed behavior) while others do not (somatic traits), or where cross-sex shifts are found in some traits in females (digit ratio) but not in males, point to a possible patterning of causal pathways by trait, sex, and developmental stage.

As mentioned before, the number and extent of critical periods for prenatal sex hormone actions might be important. There is a growing theoretical suggestion that males may have more than one critical period (e.g., prenatal, early postnatal, and pubertal), while females may have several but longer sustaining “sensitive periods” in which sex hormones and other developmental processes may act over a longer time period to influence behavioral outcomes (McCarthy et al., 2018). It is important to note that there are no longitudinal studies linking direct measures of prenatal androgens, such as amniotic levels of fetal testosterone, with later sexual orientation and cognition in humans. Such prospective studies would provide the critical test of the prenatal androgen model. Such studies will also need to control for important confounders or third factors such as genetics (e.g., genetic correlations between the traits in question over time). Such third factors might also be more important in the causal association between male sexual orientation and associated behavioral traits. One such factor is the well-known fraternal birth order effect (FBO; Blanchard, 2018). This refers to the robust finding that homosexual men have more older brothers than heterosexual men, an effect ascribed to maternal immune responses triggered by carrying successive male fetuses which affects sexual differentiation of the brain of later born males (Bogaert et al., 2018). One study has reported no significant association between FBO and spatial cognition in heterosexual and homosexual males (Rahman, 2005b; cf. Bogaert, 2003a, 2003b).

The current meta-analysis had several important limitations. Many of these are similar to those in our original meta-analysis so will not be repeated here. However, specific to the present analysis, we note that the heterogeneity between studies was high given the broad 95% confidence intervals. We have suggested that methodological variation (e.g., cognitive domain differences) is a significant contributor to this heterogeneity. Second, the number of studies for some cognitive domains included in the multivariate meta-analysis was small, which generally resulted in broad 95% confidence intervals (e.g., spatial location memory, female-favoring tasks, and male-favoring other tasks). Broad 95% confidence intervals indicate considerable uncertainty in effect sizes. Thus, more research with appropriate sample sizes is needed and this may change the conclusions. Finally, we were unable to find sufficient numbers of studies which reported within-group correlations between multiple cognitive tasks (only four studies reported the correlations). This latter point is of note for future research because having within-group correlations between tasks would permit the calculation of multivariate effect sizes (such as Mahalanobis D or other indices of multivariate distances). Such metrics would allow tests of the overall magnitude of sexual orientation differences where the groups differ along many variables of interest or where the construct is multidimensional (Del Giudice, 2013).
